# Research on a Time Difference Processing Method for RTD-Fluxgate Data Based on the Combination of the Mahalanobis Distance and Group Covariance

**DOI:** 10.3390/s23229223

**Published:** 2023-11-16

**Authors:** Na Pang, Dan Wang, Yuhan Yang, Rui Wang

**Affiliations:** College of Computer Science and Technology, Beihua University, No. 3999 East Binjiang Road, Jilin 132013, China; pangna505@163.com (N.P.); yangyuhan510@sina.com (Y.Y.); wangrui@beihua.edu.cn (R.W.)

**Keywords:** RTD-fluxgate, time difference, gross error, Mahalanobis distance, group covariance

## Abstract

During the measurement of magnetic fields, Residence Time Difference (RTD)-fluxgate sensors suffer from abnormal time difference jumps due to the random interference of magnetic core noise and environmental noise, which results in gross errors. This situation restricts the improvement of sensor accuracy and stability. In order to solve the above problems efficiently, a time difference gross error processing method based on the combination of the Mahalanobis distance (MD) and group covariance is presented in this paper, and the processing effects of different methods are compared and analyzed. The results of the simulation and experiment indicate that the proposed method is more advantageous in identifying the gross error in time difference. The signal-to-noise ratio for the time difference is improved by about 34 times, while the fluctuation of the Negative Magnetic Saturation Time (NMST) Δ*T*_NMST_ is reduced by 95.402%, which significantly reduces the fluctuation of time difference and effectively improves the accuracy and stability of the sensor.

## 1. Introduction

In recent years, Unmanned Aerial Vehicle (UAV) aeromagnetic measurement as a new aeromagnetic technology has become a hot research topic [1]. Due to the characteristics of high sensitivity, small size, low power consumption, etc., the fluxgate sensor contributes to the trend of portable measurement with UAV as a carrier [2]. In particular, the RTD-fluxgate can avoid odd harmonic interference and allows for complete symmetry of the two-axis structure of the sensing unit in terms of the measurement principle [3,4,5]. In order to better meet the requirements for UAV aeromagnetic measurement, further improving the accuracy and stability of an RTD-fluxgate has become a key research topic [6,7,8]. 

When the RTD-fluxgate is working, the magnetic core produces random magnetic noise under the action of the excitation magnetic field [9,10]. The detection circuit and time difference during transmission are easily affected by environmental noise. The above situation causes abnormal time difference jumps, reducing the stability of the RTD-fluxgate [11,12]. Therefore, it is necessary to estimate and deal with the gross error in time difference. The Laida criterion can be used if the processing method adopted includes discriminating and eliminating gross errors. However, this method is only suitable for cases with small measurement times and low processing accuracy [13]. When the data contain only a single outlier, the Grubbs criterion can be used more easily and effectively, but it cannot deal with a large number of outliers [14,15]. When the comparatively large amount of data does not follow a normal distribution, using the Chauville criterion reduces the accuracy of the measurement [16]. This shows that the scope of application for different criteria is diverse. The direct elimination of gross errors can lead to important data information being removed. If we use the moving average filtering method, the random fluctuation caused by noise can be effectively suppressed, but the selection of window parameters directly affects the smoothing effect on the data. When larger window parameters are selected, the determinacy of the high-frequency variation is weakened on average. However, when smaller window parameters are selected, the low-frequency random fluctuation is not averaged [17]. At the same time, the amount of data is reduced due to the added windows processing. It can be seen that the methods above have limitations when dealing with the gross error in time difference.

Because the MD is sensitive to data outliers, it is suitable as a statistic for measuring outliers [18,19]. The MD of the time difference is regarded as the standard, so the gross error in the time difference can be accurately estimated. In addition, covariance is the measure of the degree of deviation from its mean. Therefore, in order to eliminate the gross errors in real-time dynamic magnetic field measurement using RTD-fluxgate, this article proposes a method for processing time difference gross errors by combining MD with group covariance as weights cooperating with mean values. This method can reduce the time difference fluctuation caused by random noise, improve the stability of RTD-fluxgate, and better process the time difference dynamically in real time.

## 2. Structure and Working Principle of RTD-Fluxgate

**Figure 1:**
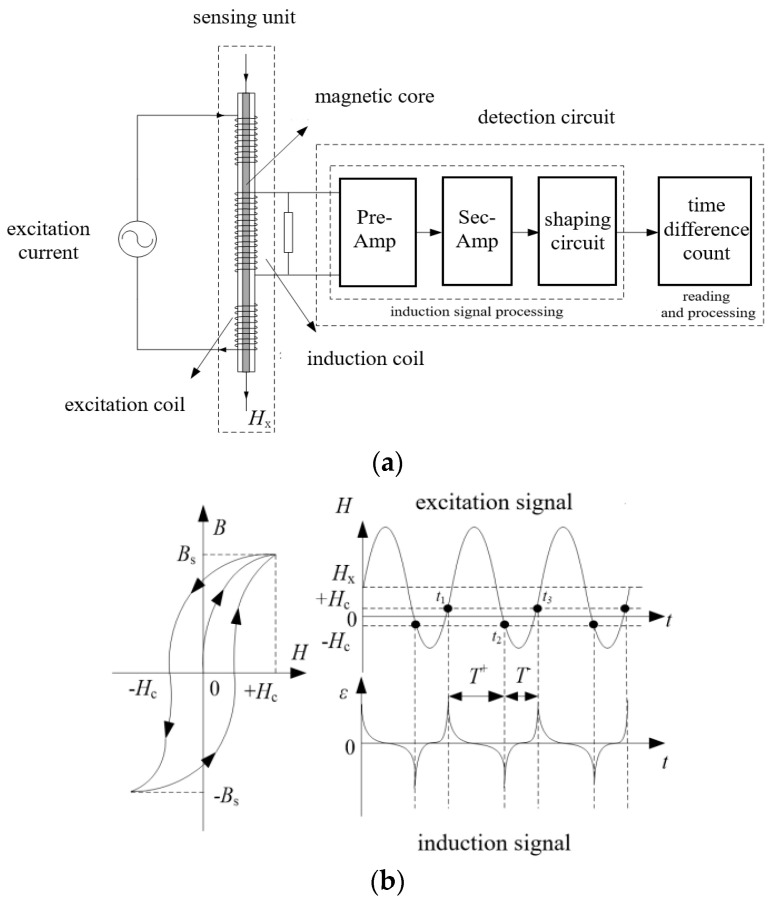
Schematic diagrams of the RTD-fluxgate structure and its working principle. (**a**) The structure of an RTD-fluxgate sensor; (**b**) A schematic diagram of the RTD-fluxgate system’s working principle.

The RTD-fluxgate sensor is mainly composed of a sensing unit and a detection circuit, as is shown in Figure 1a. When it is working, the periodic alternating excitation current is input to both ends of the excitation coil of the sensing unit in order to generate an excitation magnetic field, where the magnetic core is magnetized to a bidirectional supersaturated state. As is shown in Figure 1b, after the excitation magnetic field is modulated by the hysteresis loop of the magnetic core, a pulse shape induction signal is generated through the induction coil. We can obtain the target magnetic field *H*_x_ by determining the time difference between the positive and negative pulses of the induction signal relative to the magnetic saturation [20,21]. 

**Figure 2:**
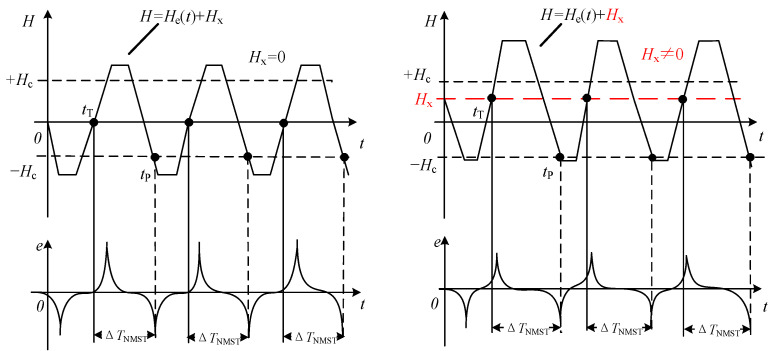
Diagram of the NMST readout strategy.

In order to minimize the influence of noise on the detection of and reduce the uncertainty in the time difference, we use the excitation signal and the output pulse signal, combined, to find the Negative Magnetic Saturation Time (NMST) ∆*T*_NMST_, which is the quantity used to measure the target magnetic field *H*_x_ [22,23,24]. The schematic diagram for the NMST reading technology is shown in Figure 2. When the magnetic core becomes saturated, an output pulse signal is generated on the induction coil; the transition time when the excitation signal amplitude is zero is used as the reference time, *t*_T_; and when the applied magnetic field exceeds the coercive field, −*H*_c_, the induced voltage produces a negative pulse at *t*_P_.

When the trapezoidal excitation field *H*_e_(*t*) is used, assuming the total time of the slope within a cycle is $t^{\prime }$, the stable state time is $t^{\prime \prime }$. *T*_e_ is the excitation period, and *f* is the excitation frequency, so $t^{\prime }$ + $t^{\prime \prime }$ = *T*_e_. The slope of the trapezoidal excitation is ±*a*, and the stable amplitude is ±*H*_m_. The expression is as follows:

(1) $$H_{e}(t)=\left\{\begin{array}{l} at\;NT_{e}-\frac{t^{\prime }}{4}<t<NT_{e}+\frac{t^{\prime }}{4}\\ H_{m}\;NT_{e}+\frac{t^{\prime }}{4}<t<NT_{e}+\frac{T_{e}}{2}-\frac{t^{\prime }}{4}\\ -at\;NT_{e}+\frac{T_{e}}{2}-\frac{t^{\prime }}{4}<t<NT_{e}+\frac{T_{e}}{2}+\frac{t^{\prime }}{4}\\ -H_{m}\;NT_{e}+\frac{T_{e}}{2}+\frac{t^{\prime }}{4}<t<NT_{e}+T_{e}-\frac{t^{\prime }}{4} \end{array}\right.$$

The relationship between the excitation field *H*_e_(*t*) and the target field *H*_x_ is as follows:

(2) $$\begin{array}{cc} t_{T}: & \alpha t_{T}=0 \end{array}$$

(3) $$\begin{array}{cc} t_{P}: & H_{x}-\alpha (t_{P}-\frac{T_{e}}{2})=-H_{c} \end{array}$$

Deduced using Equations (2) and (3):

(4) $$t_{T}=0$$

(5) $$t_{P}=\frac{\left(H_{c}+H_{x}\right)}{\alpha }+\frac{T_{e}}{2}$$

The time difference ∆*T*_NMST_ between *t*_P_ and *t*_T_ is defined by the Negative Magnetic Saturation Time, which is given by the following:

(6) $$\Delta T_{\mathrm{NMST}}=t_{P}-t_{T}$$

The expression of the relationship between ∆*T*_NMST_ and *H*_x_ is as follows:

(7) $$\Delta T_{\mathrm{NMST}}=t_{P}-t_{T}=\frac{\left(H_{c}+H_{x}\right)}{4H_{m}f_{}}+\frac{T_{e}}{2}$$

The sensitivity *S*_NMST_ expression found using the NMST reading strategy is as follows:

(8) $$S_{\mathrm{NMST}}=\frac{\partial \Delta T_{\mathrm{NMST}}}{\partial H_{x}}=\frac{1}{\alpha_{}}=\frac{T_{e}}{4H_{m}}=\frac{1}{4H_{m}f}$$

## 3. Discriminating and Processing Methods for Gross Error in Time Difference

The detection circuit in an RTD-fluxgate sensor adopts certain measures to suppress fluctuation and utilizes the NMST readout strategy to reduce the influence of induced signals. However, due to random external interference and other comprehensive factors from the environment, the original time difference still has certain fluctuation, and even some individual values fluctuate greatly, which causes a decrease in sensor stability. Therefore, it is necessary to estimate the gross error accurately and replace it with the effective value after time difference processing. Due to the sensitivity of the MD to anomalous values, the MD is regarded as a test statistic for the time difference; it can accurately estimate whether the time difference is interfered by noise or not. The covariance of the time difference can measure the deviation degree from its mean. The MD of the time difference data combined with the covariance of the corresponding group as the weight cooperate with the mean value replaces the gross time difference and reduces the impact of random fluctuation. 

### 3.1. Time Difference Gross Error Discrimination Method Based on the Mahalanobis Distance

The Mahalanobis distance is the closest distance between a single sample and the “center of gravity” of a sample set [25]; this can help calculate the similarity of two unknown sample sets and overcome the defect of Euclidean distance which lacks the overall sample to affect the distance. Considering the relationship between various features, the MD is sensitive to abnormal values [26,27,28]. For a sample set *x*_i_(=1,… n.), the *D*_m_ expression of the MD between two samples, *x*_i_ and *x*_j_, is expressed as follows:

(9) $$D_{i}=\sqrt{{\left(x_{i}-x_{j}\right)}^{T}\cdot \Sigma^{-1}\cdot (x_{i}-x_{j})}$$

Because of the sensitivity of the MD to anomalous values, it is suitable to be used as a statistic to measure anomalous values. The MD from the time difference to the total data is used as the standard to estimate the gross error. First, the time difference ∆*T*_NMST_ is read, and then the Mahalanobis distance *D*_i_ of each time difference ∆*T*_NMST(i)_ to the overall data is computed. Then, the time difference ∆*T*_NMST(i)_ whose MD is less than the threshold *d* is chosen to form a new time difference sequence *N*_1_.

If the average value $\bar{D_{i}}$ is taken as the threshold *d*, the selected threshold value *d* results in a large amount of the gross time difference being ignored, and it cannot be discriminated. If the minimum value of the time difference min (*D*_i_) is selected, the effective time difference is discriminated. Because the time difference’s Mahalanobis distance approximately obeys the chi-squared distribution with the degree of freedom P [27], the chi-squared distribution value $X_{P}^{2}(\partial )$ can be selected as the threshold value *d*. When the value *D*_i_ of the time difference Δ*T*_NMST(i)_ is larger than $X_{P}^{2}(\partial )$, the Δ*T*_NMST(i)_ is considered to be the gross error. The discriminant expression is shown in Formula (10).

(10) $$D_{i}>X_{P}^{2}(\partial )$$

**Figure 3:**
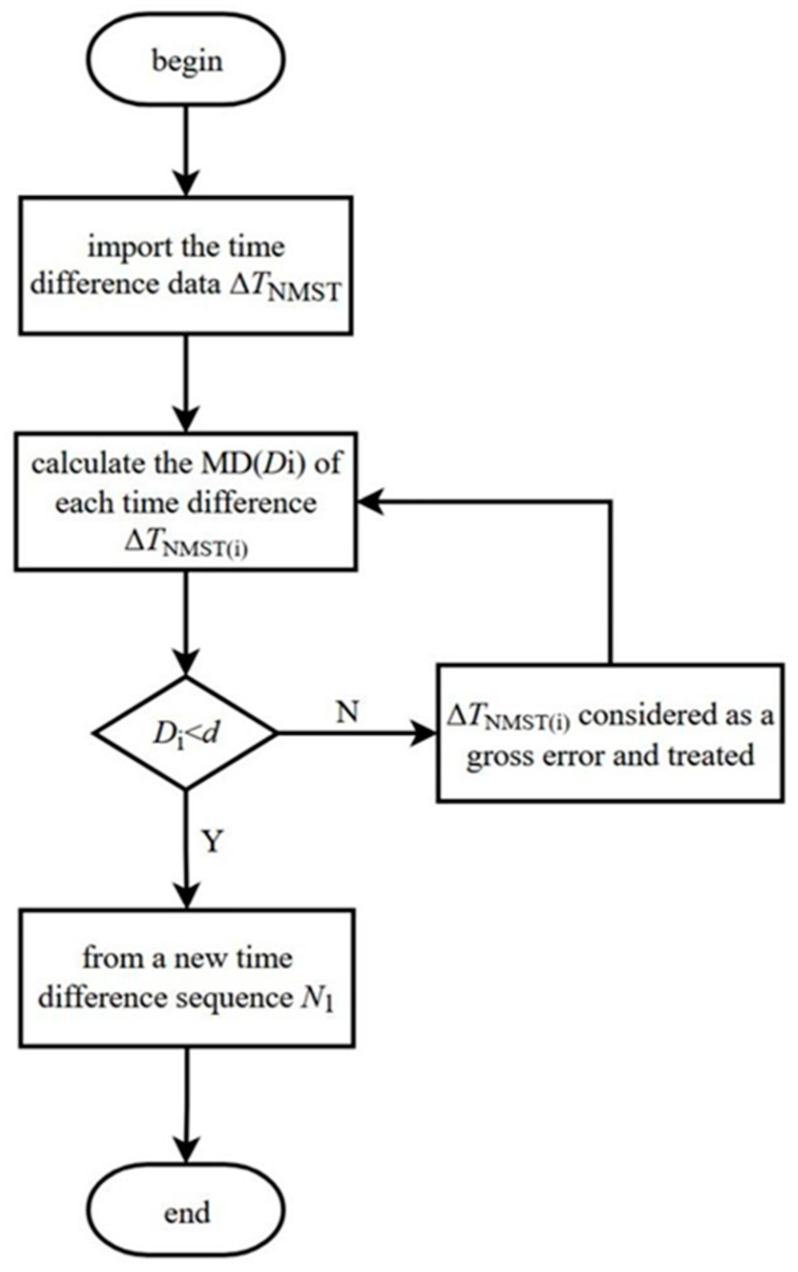
The flow chart for time difference gross error discrimination based on the MD.

In the equation above, the parameter ∂ represents the confidence level and $X_{P}^{2}(\partial )$ represents the chi-squared distribution value. In this article, the confidence probability P = 97.5% is selected. There are two variables in the formula; in the corresponding table with *α* = 0.975, *n* = 2, the value $X_{P}^{2}(\partial )$ is 0.051. The process of time difference gross error discrimination is shown in Figure 3.

### 3.2. Time Difference Gross Error Processing Algorithm Combined with the Mahalanobis Distance and Group Covariance

As seen above, the MD can be used as a statistic for outliers and it can estimate the gross error in the time difference accurately. While the data contain useful information, they need to be processed. As described in Equation (8), the MD can be understood as the degree of difference between two random variables which obey the same distribution and have a covariance matrix of Σ (Σ represents the overall covariance matrix of the sample). 

In order to better reflect the variation in the original time difference, this paper divides the time difference data into groups to obtain the covariance of each group. The MD for the time difference data combined with the covariance of the corresponding group as the weight cooperates with the mean value, with which gross time difference is replaced. The above processing can reduce random fluctuation and is suitable for the real-time dynamic processing of time difference data. Therefore, a time difference gross error processing method based on the combination of the MD and group covariance is presented in the paper. The distance between two samples (*x*_i_ − *x*_j_) in Equation (9) is replaced by the distance from the Δ*T*_NMST(i)_ to the mean value $\bar{\Delta T_{\mathrm{NMST}(i)}}$, which can be expressed as follows:

(11) $$D_{i}=\sqrt{{\left(\Delta T_{\mathrm{NMST}(i)}-\bar{\Delta T}\right)}^{T}\cdot C^{-1}\cdot (\Delta T_{\mathrm{NMST}(i)}-\bar{\Delta T})}$$

From the above equation, the distance between the time difference ∆*T*_NMST(i)_ and the mean value $\bar{\Delta T_{\mathrm{NMST}(i)}}$ is $\sqrt{C}\cdot D_{i}$. In this paper, the time difference ∆*T*_NMST(i)_ is divided into *n* groups, for which the covariance *C*_n_ of each group can be calculated. Therefore, the *D*_i_ of ∆*T*_NMST(i)_ is multiplied by the corresponding covariance *C*_n_ of the number *n* group of time difference data as the weight. The number *i* time difference ∆*T*_NMST(i)_ processed by this algorithm is *F*_i_, shown in Equation (12):

(12) $$F_{i}=\Delta T_{\mathrm{NMST}(i)}\pm (\sqrt{C_{n}}\cdot D_{i})\;(I=1,\;\ldots ,\;n)$$

**Figure 4:**
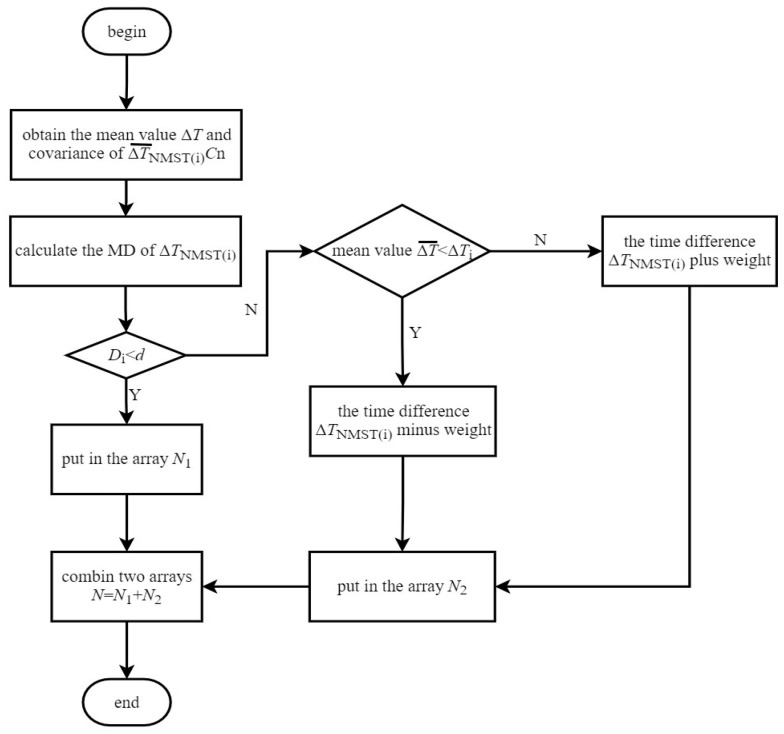
The flow chart of the processing algorithm.

The flow chart of the time difference gross error processing method proposed in this paper is shown in Figure 4. First, the mean value of the time difference data is obtained, and the data are divided into *n* groups to calculate the covariance *C*_n_ of each group. Then, *D*_i_ of ∆*T*_NMST(i)_, for calculating time difference, is cyclically traversed to determine whether *D*_i_ is less than the threshold *d* or not. 

If *D*_i_ < *d*, the time difference ∆*T*_NMST(i)_ is normal, and this value is stored in the array *N*_1_ to continue traversing the time difference ∆*T*_NMST(i+1)_; if *D*_i_ ≥ d, the time difference ∆*T*_NMST(i)_ is considered to be a gross error and needs to be processed. When the time difference ∆*T*_NMST(i)_ is greater than the mean value of the data $\bar{\Delta T_{\mathrm{NMST}}}$, the ∆*T*_NMST(i)_ is subtracted from the square root of the covariance *C*_n_ of the corresponding group of time difference data, multiplied by the time difference ∆*T*_NMST(i)_. When the time difference ∆*T*_NMST(i)_ is smaller than the mean value $\bar{\Delta T_{\mathrm{NMST}}}$ of the data, ∆*T*_NMST(i)_ is added to the square root of the covariance *C*_n_ of the corresponding group of time difference data, multiplied by the time difference ∆*T*_NMST(i)_.

In this process, when each time difference gross error processing finishes, it is stored in the array *N*_2_ until all the gross error processing is completed. The time difference data *F*_i_ in *N*_2_ and the normal time difference array *N*_1_ form a new time difference array *N*.

The above processing can reduce random fluctuation and is suitable for the real-time dynamic processing of time difference data. Meanwhile, the algorithm is fast, simple, and easy to implement, which enables it to effectively meet the real-time dynamic processing requirements and improves the stability of the RTD-fluxgate system.

## 4. Simulation Data Analysis

In order to verify the effectiveness of the algorithm proposed in this paper, the Matlab Simulink random signal module is used to simulate the time difference in RTD-fluxgate; three groups of time difference data are obtained, with 1000 time differences in each group, and with different mean values and variance. The Laida criterion, moving average filtering, and the algorithm proposed in the paper are, respectively, used for processing. The statistical values resulting from the different processing methods are compared, as shown in Table 1.

As seen in Table 1, the mean values after processing using the three methods remain basically unchanged. The deviation and the fluctuation in the time difference are reduced by 2.034% and 1.797%, respectively, after processing using the Laida criterion. Through the use of moving average filtering, the variance of time difference is reduced by 98.428% and the fluctuation decreases by at least 88.963%. However, after using the algorithm proposed in this paper, the variance of the time difference is reduced by 99.392% and the fluctuation decreases by at least 90.896%. It can be concluded that the method of combining the MD and group covariance has a better processing effect.

The traditional signal-to-noise ratio reflects the ratio between the energy of the signal and the noise, but it can also evaluate the effect of noise processing. In order to better evaluate the processing effect of the time difference gross error, we consider the working principle of RTD-fluxgate and the particularity of the physical quantity of the magnetic core negative saturation time Δ*T*_NMST_. In this paper, the ratio of the core negative saturation time mean value $\bar{\Delta T_{\mathrm{NMST}}}$ and the noise-induced uncertainty *δ*(Δ*T*_NMST_) are defined as the time difference signal-to-noise ratio Δ*T*_SNR_, as is shown in Equation (13), and this parameter is used to quantitatively evaluate the processing effect of the time difference coarse error.

(13) $$\Delta T_{\mathrm{SNR}}=\frac{\bar{\Delta T_{\mathrm{NMST}}}}{\delta (\Delta T_{\mathrm{NMST}})}$$

When the observation time is *T*_ob_ and the number of cycles collected is *N*, then

(14) $$T_{\mathrm{ob}}=NT_{e}=N(\Delta T_{\mathrm{PMST}}+\Delta T_{\mathrm{NMST}})$$

In this formula, Δ*T*_PMST_ and Δ*T*_NMST_ represent the residence time of the magnetic core in the positive and negative saturation state, respectively. Since the residence time distribution of the positive and negative saturated states is the same and uncorrelated, and the standard deviation is approximately equal, then

(15) $$\sigma \Delta T_{\mathrm{NMST}}\approx \sigma \Delta T_{\mathrm{PMST}}$$

The uncertainty *δ*(Δ*T*_NMST_) of the magnetic core negative saturation time Δ*T*_NMST_ is shown in Equation (16):

(16) $$\delta (\Delta T_{\mathrm{NMST}})=\frac{\sigma \Delta T_{\mathrm{NMST}}}{\sqrt{N}}$$

In this formula, σΔ*T*_NMST_ is the standard deviation of the Δ*T*_NMST_ distribution. By substituting Equation (16) into Equation (13), the expression for the time difference signal-to-noise ratio Δ*T*_SNR_ can be obtained as follows:

(17) $$\Delta T_{\mathrm{SNR}}=\frac{\bar{\Delta T_{\mathrm{NMST}}}}{\delta (\Delta T_{\mathrm{NMST}})}=\frac{\bar{\Delta T_{\mathrm{NMST}}}}{\sigma \Delta T_{\mathrm{NMST}}}\sqrt{N}$$

The first set of simulated time difference data is selected, and the Δ*T*_SNR_ processing using the three different methods is shown in Table 2. It can be seen from the table that the Δ*T*_SNR_ obtained using the method based on the combination of the MD and group covariance is obviously higher than that obtained using the other two methods, so this method is suitable for processing the coarse value of the time difference.

## 5. Experiment and Preliminary Results

Due to the existence of geomagnetic fields and external interference from magnetic fields, the test was greatly influenced. In order to improve the stability and accuracy of the test, a Helmholtz coil was placed in the middle area of the electromagnetic shielding cylinder made of a five-layer permalloy in an electromagnetic shielding room of the National Geophysical Exploration Instrument Engineering and Technology Research Center of Jilin University. The RTD-fluxgate sensor was made by the Key laboratory of geophysical exploration equipment and it was placed in the uniform region of the Helmholtz coil; the measured magnetic field *H*_x_, which was parallel to the core axis, was applied to the sensing unit.

**Figure 5:**
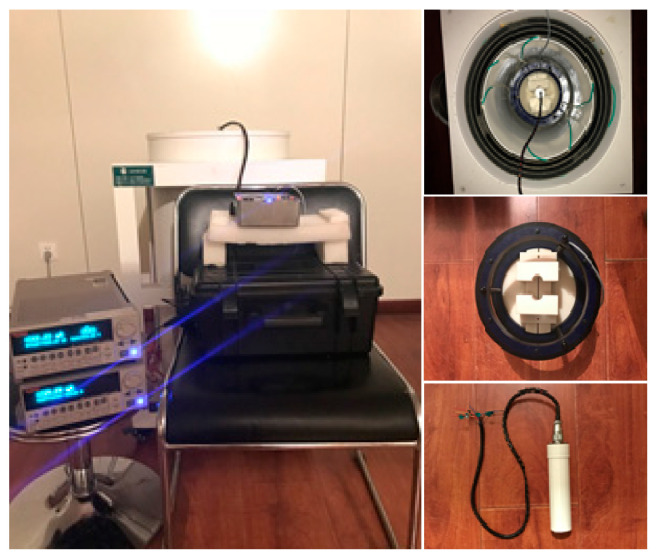
Instrument connection and test environment.

The precision current sources of KEITHLEY 6221 were utilized in this experiment to drive the excitation coil of the RTD-fluxgate sensor to generate an excitation magnetic field. The excitation magnetic field waveform adopted a trapezoid-wave magnetic field with high time difference stability. When the frequency *f* = 30 Hz and the current *I* = 60 mA, the induction voltage produced by the induction coil pass through, in turn, the preamplifier circuit Pre-Amp, the secondary amplifier circuit Sec-Amp, and the shaping circuit. The shaping circuit consists of an addition circuit, a bias voltage generating circuit, and a hysteresis comparison circuit. When the induction voltage passes through the hysteresis comparison circuit, if using a threshold slightly lower than the peak value of the induction voltage, the shaping circuit obtains a rectangular wave with a duty cycle which varies with the information of the measured magnetic field *H*_x_. This rectangular signal is input to the CH1 channel of the FPGA logic signal processor. The excitation current source is adjusted to generate a synchronous trigger pulse. When the excitation voltage amplitude is zero, this is set as a trigger point, and the synchronous trigger pulse is input to the CH2 channel of the FPGA. It uses two-channel signals to count the time difference, in which the counting frequency *f*_c_ of FPGA is 100 MHz. The number of time points *N* is converted into the time difference Δ*T*_NMST_ and transmitted to STM32 for storage. The relevant instrument connection and test environment are shown in Figure 5.

By changing the current at both ends of the Helmholtz coil, *H*_x_ is gradually moved through the range of −52,500~+52,500 nT and the least squares method is used to fit it linearly. When the sum of squared deviations from each data point to the fitting curve is minimized, the relationship between Δ*T*_NMST_ and *H*_x_ is obtained as follows:

Δ*T*_NMST_ = 0.008039 × *H*_x_ + 1.612 × 10^4^ (18)

**Figure 6:**
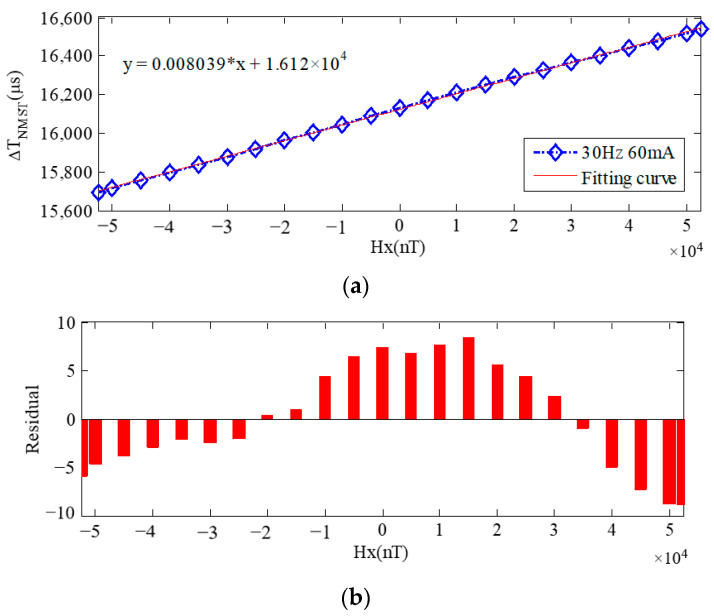
Fitting linearity deviations of Δ*T*_NMST_ with *I*= 60 mA and *f* = 30 Hz. (**a**) Fitting curve of Δ*T*_NMST_; (**b**) deviations of Δ*T*_NMST_.

As in the equation above, the sensitivity of the sensor is *S*_NMST_ = 0.008039 μs/nT. The relationship between the output Δ*T*_NMST_ and the measured magnetic field *H*_x_, as well as the linear deviations, are shown in Figure 6.

Figure 6a shows the fitting curve of Δ*T*_NMST_. According to the linear relationship, the sensitivity of the sensor *S*_NMST_ is 0.008039 μs/nT. Figure 6b shows the linear deviations of Δ*T*_NMST_. When *H*_x_ = +52,500 nT, Δ*T*_NMST_ is 16,517.17 μs and the maximum residual value between the measured results and the fitting curve is 8.87 μs. Therefore, the linear error (relative error) of the sensor is γ < ±0.054%. It can be seen from the figure that the RTD-fluxgate system possesses good linearity in the whole range of measurement.

**Figure 7:**
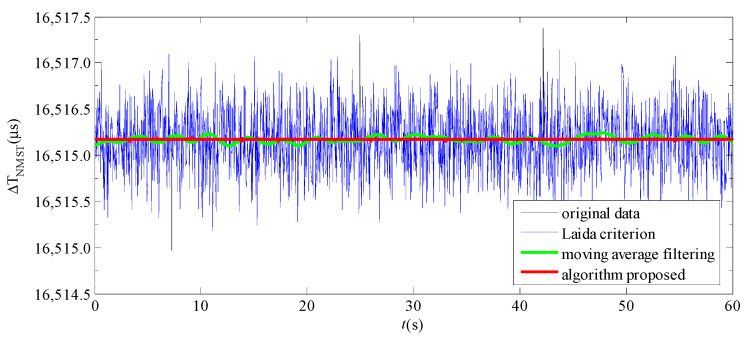
Comparison diagram of Δ*T*_NMST_ processed using different methods.

To verify the effectiveness of the proposed algorithm in this paper, when *H*_x_ = +50,000 nT and the observation time *t* = 1 h, the Δ*T*_NMST_ are divided into 30 groups and averaged. Then, the Δ*T*_NMST_ are processed using the Laida criterion, moving average filtering, and the algorithm proposed in this paper, respectively. Because the observation time is longer, the data of the time difference Δ*T*_NMST_ are larger. We only present the Δ*T*_NMST_ processed using different methods for 60 s among 1 h, as shown in Figure 7. 

**Figure 8:**
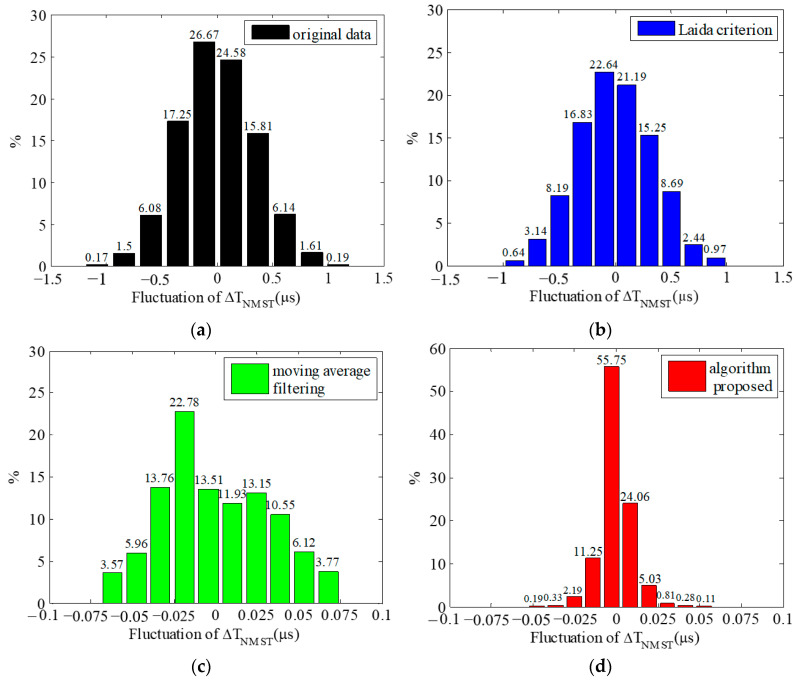
Histograms of Δ*T*_NMST_ fluctuation distribution using different methods. (**a**) Fluctuation in Δ*T*_NMST_ without processing; (**b**) fluctuation in Δ*T*_NMST_ after processing using the Laida criterion; (**c**) fluctuation in Δ*T*_NMST_ after processing using moving average filtering; (**d**) fluctuation in Δ*T*_NMST_ after processing using the combination of the MD and covariance.

As is illustrated in Figure 7, after being processed using the proposed algorithm, the stability of Δ*T*_NMST_ is obviously higher than that of the other two methods. The statistical value of Δ*T*_NMST_ processed using different methods is shown in Table 3. According to the table, with the mean of Δ*T*_NMST_ unchanged, the time difference signal-to-noise Δ*T*_SNR_ obtained using the method of combining the MD and group covariance is 47.776, which is about 34 times higher than that without any processing and is significantly higher than the other two methods. The standard deviation is reduced from 0.340 μs to 0.010 μs, which effectively decreases the time difference fluctuation caused by noise. The histogram of the Δ*T*_NMST_ fluctuation distribution after being processed using the different methods is further calculated, as shown in Figure 8.

It can be seen from the above figure that the distribution of the Δ*T*_NMST_ fluctuation is approximately a normal distribution, and the percentage of the Δ*T*_NMST_ fluctuation greater than 1 μs is 0.360%. In Figure 8a, the fluctuation in Δ*T*_NMST_ is 2.414 μs without processing. In Figure 8b, it is 1.995 μs after processing using the Laida criterion. In Figure 8c, it is 0.144 μs after processing using moving average filtering. In Figure 8d, it is 0.111 μs after processing using the combination of the MD and group covariance. This comparison indicates that the fluctuation in the Δ*T*_NMST_ after using the proposed method is reduced by 95.402% compared with that without any processing, which significantly reduces the fluctuation in the Δ*T*_NMST_ compared with other methods, so the stability of the RTD-fluxgate system can be improved.

## 6. Conclusions

On the basis of describing the structure and working principle of RTD-fluxgate, it is known that the time difference detection is affected by external interference and environmental factors, which causes coarse error in the time difference data and reduces the stability of the sensor. In this paper, a processing method is proposed to deal with the gross error in time difference by combining the MD with group covariance as weights cooperating with mean values. The results of our simulation and experiment indicate that the proposed method is more advantageous in identifying the time difference gross error: the signal-to-noise ratio of the time difference is improved by 34 times and the fluctuation in the Δ*T*_NMST_ is reduced by 95.402%. The processing effect is better than that of other methods, and the influence of random interference on time difference detection is reduced. Meanwhile, the algorithm is fast, simple, and easy to implement, which can effectively meet the real-time dynamic processing requirements and improve the stability of RTD-fluxgate.

## Figures and Tables

**Table 1:** The statistical values from the different processing algorithms.

First Set of Data				Second Set of Data			Third Set of Data		
	Mean Value	Variance (≈)	Fluctuation	Mean Value	Variance (≈)	Fluctuation	Mean Value	Variance (≈)	Fluctuation
original data	804.545	30.911	32.280	500.000	10.178	19.881	200.000	20.573	28.042
Laida criterion	804.524	29.938	31.700	499.857	9.971	18.023	200.083	19.765	25.435
moving average	804.569	0.324	3.299	499.905	0.160	2.194	200.035	0.222	2.591
algorithm in paper	804.546	0.126	2.543	499.910	0.037	1.771	200.022	0.125	2.553

**Table 2:** The Δ*T*_SNR_ of the simulated time difference using different processing methods.

	Original Data	Laida Criterion	Moving Average	Algorithm in Paper
$\bar{\Delta T_{\mathrm{NMST}}}$ (μs)	804.545	804.524	804.569	804.546
σΔ*T*_NMST_ (μs)	5.682	5.472	0.569	0.355
Δ*T*_SNR_	4.477	4.649	44.714	71.667

**Table 3:** Statistical values of Δ*T*_NMST_ using different processing methods.

	Mean Value (μs)	Fluctuation (μs)	Standard Deviation (μs)	Δ*T*_SNR_
original data	16,516.169	2.414	0.340	1.349
Laida criterion	16,516.168	1.995	0.335	1.370
moving average filtering	16,516.190	0.144	0.032	14.246
algorithm in this paper	16,516.168	0.111	0.010	47.776

## Data Availability

The data presented in this study are available on request from the corresponding author.
